# Bioactive Profiling and Anti-Hyperglycemic Potential of *Berberis nummularia* Bunge: Role of Polyphenols and α-Amylase Inhibition

**DOI:** 10.3390/foods14244180

**Published:** 2025-12-05

**Authors:** Buhailiqiemu Abudureheman, Lin Chen, Jianlin Zhang, Shuai Zhu, Jinjuan Wang, Junli Huang, Chaoying Xie, Haibo Pan, Xingqian Ye

**Affiliations:** 1 Center for Experimental Instruction in Food Safety and Nutrition, Xinjiang Institute of Technology, Aksu 843000, China; buhalqam.a@163.com (B.A.); nei3677585@163.com (L.C.); zjl950702@163.com (J.Z.); z6988752@163.com (S.Z.); 19190574829@163.com (J.W.); mengduud@163.com (J.H.); chaoyingxie@163.com (C.X.); 2 Zhejiang Key Laboratory of Agri-Food Resources and High-Value Utilization, Fuli Institute of Food Science, College of Biosystems Engineering and Food Science, Zhejiang University, Hangzhou 310058, China; 3 Innovation Center of Yangtze River Delta, Zhejiang University, Jiaxing 314102, China

**Keywords:** *Berberis nummularia*, phytochemical constituents, antioxidant activity, α-amylase inhibition, hypoglycemic effect

## Abstract

The *Berberis nummularia* fruit is rich in polyphenols and which are associated with the inhibition of carbohydrate-digesting enzymes. However, the phytochemical compositions, antioxidant strength, and the ability of the fruits on the inhibition of α-amylase to control postprandial blood glucose remained elusive. In this study, therefore, different concentrations of ethanol were used in ultrasound processing at 70 °C for 1 h to obtain the crude polyphenol of *B. nummularia* fruit (CPB) and obtain the purified polyphenol (PPB) using AB-8 macroporous resin. After this, the polyphenolic constituents within PPB were identified using LC-MS/QTOF and investigated for anti-hyperglycemic properties by sucrose loading test. The results showed that the optimal extraction yield (44.32 ± 2.08%) of CPB was achieved with 30% ethanol and the PPB from CPB was reached at 71.88 ± 2.74%. A total of 30 polyphenols including 13 phenolic acids, 13 flavonoids, 3 benzaldehyde derivatives, and 1 aromatic acid were identified, in which the caffeic acid had the highest content (426.20 ± 0.18 ng/mg). The PPB displayed potent α-amylase inhibitory activity with an IC_50_ value of 69.91 μg/mL and kinetic analysis via Lineweaver–Burk double reciprocal plots confirmed a non-competitive inhibition mechanism. Moreover, at an administration dose of 100 mg/kg body weight (BW), PPB significantly reduced blood glucose levels by 13.75 ± 0.87% and exerted a marked ameliorative effect on postprandial hyperglycemia in vivo. Therefore, these findings provide a foundation for considering PPB as a beneficial functional food ingredient and a potential dietary supplement for the management of postprandial hyperglycemia.

## 1. Introduction

Diabetes, a widespread metabolic and endocrine disorder, has become a global health crisis affecting over 536 million people worldwide, with the number projected to surge to 783 million by 2045 [1]. Current diabetes treatments primarily rely on insulin or medications such as acarbose and voglibose [2]. However, these drugs can cause adverse effects including gastrointestinal distension, abdominal cramps, and potential hepatotoxicity [3]. Research reveals that plant-based bioactive compounds inhibiting digestive enzymes such as α-amylase, which regulates postprandial carbohydrate absorption and delays glucose uptake, represent a safe and viable therapeutic strategy for T2DM [4,5], with gentle but long-lasting hypoglycemic properties [6]. So, researchers are dedicated to exploring safe and effective natural bioactive compounds for the prevention and intervention of T2DM.

Polyphenols are a class of natural antioxidants widely distributed in plants, exhibiting physiological activities such as antioxidant, anti-inflammatory, and glucose-regulating effects. They can ameliorate T2DM through multiple mechanisms, including inhibiting α-amylase activity to reduce postprandial glucose absorption, protecting pancreatic β-cells from oxidative damage, enhancing insulin sensitivity, and regulating gut microbiota balance [7]. According to Hu et al. [8], polyphenolic extracts from walnut green husks demonstrated significant inhibition of α-glucosidase and α-amylase. Concurrently, these compounds enhanced glycogen synthesis in insulin-resistant HepG2 cells and improved glucose uptake in both 3T3-L1 adipocytes and the aforementioned HepG2 cells. Meanwhile, the hypoglycemic effect of a lotus seed skin extract (LSSE) was evidenced by significantly lowered fasting blood glucose and a reduced area under the glucose tolerance curve (AUC) in diabetic mice, with the efficacy being clearly dose-dependent [9]. Consequently, polyphenols have emerged as a research hotspot for the development of functional foods or natural anti-diabetic supplements. Maximizing the extraction efficiency of polyphenols from plant resources is essential for their practical application. Phenolic compounds are typically extracted using various solvents; Ismail reported that solvents of different concentrations exhibited significant differences in the extraction efficiency of phenolics and the antioxidant capacity of baobab (*Adansonia digitata*) fruit pulp [10]. The rational selection of an appropriate solvent system is critical for the efficient extraction and accurate quantification of total phenolic content (TPC), total flavonoid content (TFC), and various antioxidant compounds [11]. Chaanani et al. and Nguyen et al. [12,13] demonstrated that ethanol serves as an optimal solvent for efficient phytochemical extraction.

*Berberis nummularia* Bunge distributed in Xinjiang, is a culturally significant tree with a long history of traditional use in nutrition and medicine [14]. The fruit of *B. nummularia* is rich in organic acids, fiber, fat, soluble sugar, titratable acid, and minerals and polyphenols [15]. Buhailiqiemu et al. [15] reported that *B. nummularia* fruits are characterized by a significant antioxidant potential because of the high content of polyphenols. Nevertheless, the precise composition of *B. nummularia* fruits and the inhibitory effects on α-amylase activity remain incompletely elucidated.

Therefore, this study hypothesizes that ethanol solutions can enhance the extraction efficiency of polyphenols from *B. nummularia* fruits, and that the purified polyphenol extract (PPB) exhibits strong α-amylase inhibitory activity and attenuates postprandial hyperglycemia in vivo by delaying glucose absorption. The objectives of this study were to (1) characterize the polyphenolic composition and concentrations of *B. nummularia* fruit extracts using UPLC-QE, (2) optimize ethanol concentration to maximize polyphenol extraction yield and antioxidant potential, and (3) evaluate PPB’s α-amylase inhibitory activity and its therapeutic potential against postprandial hyperglycemia in vivo. This comprehensive study provides a theoretical foundation for optimally utilizing *B. nummularia* as a natural hypoglycemic agent and thus paves the way for developing related functional foods.

## 2. Materials and Methods

### 2.1. Materials and Chemicals

*B. nummularia* fruits were harvested from Aksu, Xinjiang, respectively. Gallic acid, ascorbic acid, and trolox were procured from Yuanye Biotechnology Ltd. (Shanghai, China). Ethanol and other chemicals were purchased from Sinopharm Chemical Reagent Co. (Shanghai, China). Analytical and chromatographic-grade chemicals were utilized for the study.

### 2.2. Moisture Content (MC)

The sample powder of 5 g (*B. nummularia* fruits) was dried at 105 °C in an oven (PH070A, Yiheng Ltd., Shanghai, China) at 60 min intervals for three replicates until it achieved a consistent weight. The dried samples were then removed and cooled in a desiccator containing silica gel to prevent moisture reabsorption. The fruit was re-evaluated for weight, and the MC was determined by assessing the reduction in weight concerning their original mass.

### 2.3. Extraction Yield of Polyphenol by Different Solvents

5 g of fruit powder was mixed with 100 mL of different concentrations (30%, 50%, 80%, 100%) of ethanol solutions and water. After ultrasonication at 30 °C for 30 min at 40 kHz, they were extracted for 60 min at 70 °C. Subsequently, the samples were spun at 5000 g for 15 min at 20 °C. The resulting supernatants were filtered, concentrated, and freeze-dried for 48 h at −52 °C. Each dried extract was weighted and extraction yield was calculated using Equation (1) below and kept at 4 °C [16].

(1) $$\mathrm{Extraction}\;\mathrm{yield}\;(\%)=\frac{\mathrm{dry}\;\mathrm{weight}\;\mathrm{of}\;\mathrm{extract}}{\mathrm{dry}\;\mathrm{weight}\;\mathrm{of}\;\mathrm{sample}}\;\times \;100$$

### 2.4. Determination of Polyphenol Purification Process

A 20 mL of crude polyphenol extract (CPB, 5 mg/mL) was mixed with 5 g of pretreated AB-8 macroporous resin. The mixture underwent static adsorption for 4 h in the dark at 25 °C with constant shaking (190 r/min). Following adsorption equilibrium, the filtrate was collected after filtration for the determination of polyphenol concentration [17]. The adsorption capacity and rate were calculated using Equations (2) and (3).

Following filtration, the polyphenol-saturated resin was washed with distilled water to eliminate unadsorbed impurities. Subsequent elution was carried out with 30 mL of 70% ethanol for 4 h under the same conditions (25 °C, 190 r/min) to achieve full desorption [18]. The polyphenol desorption rate was then determined via Equation (4).

(2) $$\mathrm{Adsorption}\;\mathrm{capacity}\;(\mathrm{mg}/g)\;=\frac{{(C}_{0}-{\;C}_{1})V_{1}}{m}$$

(3) $$\mathrm{Adsorption}\;\mathrm{rate}\;(\%)=\frac{\left(C_{0}-C_{1}\right)}{C_{0}}\;\times \;100$$

(4) $$\mathrm{Desorption}\;\mathrm{rate}\;(\%)=\frac{\left(C_{2}\times V_{2}\right)}{mQ}\;\times \;100$$

Here, the mathematical model incorporated the following parameters: C_0_ denoted initial polyphenolic concentration in the extract (mg/mL), C_1_ representd residual concentration post-adsorption (mg/mL), and C_2_ indicated eluted polyphenolic concentration (mg/mL). System volumes were defined as V_1_ for initial extract volume (mL) and V_2_ for eluent volume (mL). m represents wet resin quality (g) and Q represents adsorption capacity of the resin, expressed in mg/g.

For the adsorption, 5.0 g of activated AB-8 resin was mixed with 20 mL of 5 mg/mL crude polyphenol solution of *B. nummularia* fruits (CPB), which was extracted using 30% ethanol solution. The process followed adsorption method, and the polyphenol content in the supernatant was monitored at 20 min intervals [18].

To optimize the eluent concentration, a series of experiments were conducted. Six aliquots (5.00 g each) of activated AB-8 resin were loaded with CPB (20 mL, 5.0 mg/mL). The process followed adsorption method, differing only in the desorption stage, where 30 mL of ethanol at 40–90% concentration was applied to each aliquot to assess elution efficiency. The loading concentration was determined by equilibrating five 5.00 g portions of activated AB-8 resin with 20 mL of CPB at concentrations ranging from 3.0 to 7.0 mg/mL. The process followed desorption method [16].

### 2.5. Purification of CPB by Dynamic Adsorption and Desorption on AB-8 Macroporous Resin

A column (Φ4.6 cm × 45.7 cm) was wet-packed with activated AB-8 resin to a bed height of 3/5 of the column, with fractions collected in 10 mL tubes. For chromatographic separation, a 5 mg/mL CPB stock solution was prepared through dissolution and sterile filtration (0.22 μm membrane). Following this, the process was performed at ambient temperature with a peristaltic pump sustaining a 2 mL/min flow rate.

Following adsorption, hydrophilic impurities were removed by washing with deionized water, and target compounds were eluted using 70% ethanol at 1.8 mL/min [19].

### 2.6. Component Analysis

#### 2.6.1. Preparation of Fruit Samples and Polyphenol Extraction

Following a repeated extraction procedure (×3) with 0.5 mL of 30% ethanol and 0.2% VC using ultrasonication (30 min) and centrifugation (12,000 rpm, 10 min), the supernatants were collected and pooled for analysis [20].

#### 2.6.2. UPLC Conditions

Sample extracts were analyzed using a UPLC-Orbitrap-MS system equipped with a Waters HSS T3 reversed-phase column (50 × 2.1 mm, 1.8 μm) and maintained at 40 °C. The separation employed a water/acetonitrile gradient (each containing 0.1% formic acid) at a flow rate of 0.3 mL/min. The gradient elution was programmed as follows: initial ratio of 90:10 (water/acetonitrile) maintained for 0–2.0 min, linearly adjusted to 40:60 at 6.0 to 9.0 min, and then returned to the initial conditions for re-equilibration. The total run time was 12.0 min, with an injection volume of 2 μL [21].

#### 2.6.3. MS Analysis

The Q Exactive mass spectrometer (Thermo Fisher Scientific, Waltham, MA, USA) was utilized for HRMS analysis, configured with a heated electrospray ionization source in negative polarity. Key ion source parameters were set as follows: sheath gas flow rate of 50 arb (25 L/min), auxiliary gas flow rate of 15 arb (25 L/min), where “arb” denotes instrument-specific arbitrary units. The nebulizer gas temperature was maintained at 320 °C. For the full-scan mass spectrometry, the resolution was set to 60,000, while the resolution for MS/MS was 15,000. A stepped NCE gradient of 20/30/40 was employed to cover diverse fragmentation patterns of analytes. For ionization optimization, the spray voltage was adjusted to 3.8 kV in positive ion mode and −3.4 kV in negative ion mode, consistent with typical ESI voltage ranges for polar compound analysis. Xcalibur 4.1 was utilized for spectral data acquisition and TraceFinder 4.1 software was applied for subsequent data processing, including peak extraction and compound identification, followed by result export to SPSS (18.0) for further analysis [22].

QC samples: 10 μL was taken from each test sample and mixed to prepare quality control (QC) samples. In the mass spectrometry analysis sequence, one injection of QC sample was inserted every 10 test samples. The stability of the analytical instrument was assessed from QC sample data by calculating the RSD of the retention times and peak areas of target ions. The method utilized the widely accepted threshold of RSD < 15%, in accordance with common technical specifications for validating instrument performance with complex sample matrices [23].

Blank samples: The mobile phase used in this experiment was employed as the blank control, and sample injection analysis was performed to exclude contamination from the mobile phase. Each test sample was subjected to three replicate injections. The average value of the three replicate data was taken as the final quantitative result, and the relative standard deviation (RSD) of the peak area of the target analyte was calculated.

### 2.7. The TPC, TFC and the Antioxidant Activity

(1) *TPC*

Following the Folin–Ciocalteu method [24], TPC was quantified against a gallic acid standard curve (0.2–1.0 mg/L, R^2^ = 0.9960) and expressed as mg gallic acid equivalents (GAE) per 100 g sample.

(2) *TFC*

Following the method of Yang et al. [24], TFC was analyzed against a rutin standard curve (0.1–1.0 mg/L, R^2^ = 0.9967), with results given in mg rutin equivalents per 100 g of sample.

(3) *DPPH radical scavenging assay*

The DPPH radical scavenging activity was determined according to the method reported by Tian et al. [25]; a Trolox calibration curve (0.1–1.0 mg/L) with an R^2^ of 0.9994 was employed to establish the calibration curve. Based on this curve, the antioxidant activity was calculated and reported as mg of TE per 100 g sample, a common unit in food and plant extract research.

(4) *Ferric reducing antioxidant power (FRAP) assay*

Antioxidant capacity was determined by the FRAP assay [25] and referenced to a Trolox standard curve (2.00–10.00 mg/L, R^2^ = 0.9946), with results expressed as mg Trolox equivalents per 100 g sample.

### 2.8. Inhibition Assays of α-Amylase

The α-amylase inhibitory activity was assessed according to Wang et al. [26]. Briefly, 100 μL of α-amylase solution (5 mg/mL) was pre-incubated with 100 μL of PPB solutions (0.04–1 mg/mL) at 37 °C for 5 min. The reaction was initiated by adding 800 μL of starch solution, followed by incubation at 37 °C for another 5 min. It was then terminated with 1 mL of DNS reagent, diluted with 2 mL distilled water, and heated in a boiling water bath for 5 min to develop color. After cooling to room temperature, the absorbance was measured at 540 nm. The inhibition rate was calculated using negative (PBS) and positive (acarbose, 0.05–1 mg/mL) controls, following the standard DNS assay protocol using Equation (5) below.

(5) $$\%\;\mathrm{Inhibition}\;\mathrm{rate}=(1-\frac{\;{(A}_{1}-A_{2})}{A_{3}-A_{4}\;})\;\times \;100\%$$

where A_1_ denotes the sample reaction absorbance; A_2_, the sample background; A_3_, the control reaction; and A_4_, the control background. The IC_50_ value was determined as the sample concentration required for 50% enzyme inhibition, calculated from the dose–response curve.

### 2.9. In Vivo α-Amylase Inhibition Assay

#### 2.9.1. Experimental Animals

Male ICR mice (6 weeks old) were sourced from the Zhejiang Academy of Medical Sciences (Hangzhou, China), housed at 21 ± 2 °C condition under 12 h light/dark cycle, with the relative humidity of 50 ± 10%. These parameters comply with the GB 14925-2023 [27] standards for laboratory animal environments (temperature range 18–23 °C). Prior to experimentation, the mice underwent a 7 d acclimation period with free access to standard feed and water to minimize stress-related variability, in accordance with animal welfare and experimental reliability guidelines. Animal care policies and protocols used in the experiments were approved by Dr.CanBiotechnology (Hangzhou, China) Co., Ltd. (Approval No. DRK-20250407684) on 7 April 2025.

#### 2.9.2. Sucrose Loading Test

Two PPB variants with extreme in vitro IC_50_ values were chosen for the sucrose tolerance test; after 16 h of fasting, mice were stratified into water, acarbose (100 mg/kg BW), and PPB (100 mg/kg BW) groups (n = 8); all received sucrose (2 g/kg BW) 15 min later. Blood glucose was monitored for 120 min post-challenge via tail bleeding, a standard method for serial sampling in rodents. The total glycemic response, expressed as the AUC, was determined using the established trapezoidal method. Furthermore, all tested compounds demonstrated solubility in sterile water, which is essential for formulation stability and precise in vivo dosing.

### 2.10. Statistical Analysis

All quantitative results are reported as mean ± SE (dry weight) of triplicates and were analyzed by one-way ANOVA with Duncan’s test (*p* < 0.05) for mean comparisons. SigmaPlot 12.0 was used for plotting.

## 3. Results

### 3.1. Effects of Ethanol Solutions at Varying Concentrations on the Extraction Yield

**Figure 1:**
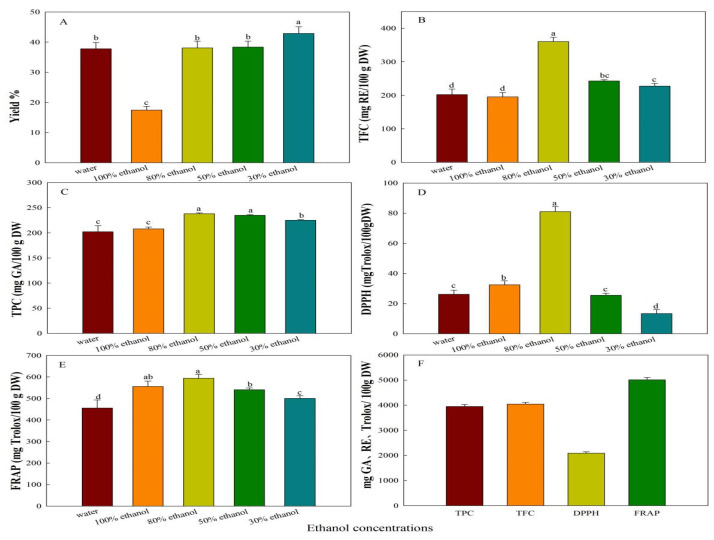
Effect of ethanol concentrations on yield (**A**), (TFC) (**B**), (TPC) (**C**), antioxidant activity as DPPH (**D**), FRAP (**E**), and the TFC, TPC, DPPH, and FRAP of purified polyphenol extracted at 30% ethanol (**F**) of *B. nummularia* fruit. Different letters above the bars denote significant differences (*p* < 0.05, Duncan’s test) in yield, TPC, TFC, DPPH, and FRAP, across ethanol concentrations.

The extraction yield of *B. nummularia* fruits in different concentrations of ethanol solutions have significant difference (Figure 1A). The extraction yield ranged from 17.46 ± 2.20% for 100% ethanol to 44.32 ± 2.08% for 30% ethanol. A progressive decrease in extraction yield was observed with rising ethanol concentration. The extract made with 30% ethanol concentration has the highest yield, followed by those using 50% and 80% and 100% ethanol solvents, the values were 38.38 ± 1.95%, 38.08 ± 2.25%, and 17.46 ± 2.20%, respectively. The study findings reveal that the extraction yield climbs as the water content in organic solvents rises. The discovery might be due to the enhanced solubility of carbohydrates and similar components in aqueous environments [28].

### 3.2. Effect of Ethanol Concentration on TPC

The total crude polyphenol content (TCPC) of *B. nummularia* fruit at the different ethanol concentrations showed a notable difference (*p* > 0.05), which varied from 202.21 ± 12.04 mg GA/100 g DW to 248.11 ± 2.42 mg GA/100 g DW, with the lowest value obtained from the water and the highest from the 80% ethanol extract (Figure 1C). The TCPC exhibited a decrease in the following sequence: 80% ethanol > 50% ethanol > 30% ethanol > 100% ethanol > water, but there is no significant difference between the TCPC of 80% ethanol and 50% ethanol, 100% ethanol and water (*p* > 0.05) (Figure 1). This results showed that moderate-concentration ethanol (80%) is more effective in extracting polyphenols from *B. nummularia* compared to other solvents. This suggests that the extraction efficiency of polyphenols is influenced by solvent polarity, with 80% ethanol likely providing an optimal balance for solubilizing a wide range of phenolic compounds; this result is the same as that of Podloucká et al. [29], who evaluated the influence of solvent choice and extraction technique on the quantification of total polyphenols in different common buckwheat varieties, resulting in the highest TPC at 80% ethanol solution. However, Umego and Barry-Ryan showed that optimum conditions for spent gin botanicals were solvent 50% ethanol [30]. Samely, the study about *Trifolium pratense* L. showed that 60% ethanol is the optimal condition for the polyphenols extraction from the flowers [31]. The species-specific response to ethanol concentration arises from differences in the composition of extractable polyphenols.

### 3.3. Effects of Solvents on TFC of B. nummularia Fruit

Figure 1B illustrates the significant differences in total flavonoid content (TFC) exhibited by different ethanol concentrations (*p* > 0.05). The TFC ranged from 195.50 ± 13.12 mg RE/100 g DW and 201.56 ± 14.04 mg RE/100 g DW for 100% ethanol and water to 380.00 ± 36.84 mg RE/100 g DW for 80% ethanol. Among the different concentrations, the 80% solvent extract showed the highest TFC value, with subsequent reductions observed in extracts prepared with 50% and 30% solvents and water and 100% ethanol. The TFC exhibited a decrease in the following sequence: 80% ethanol > 50% ethanol >30% ethanol > water > 100% ethanol. The lowest TFC values were recorded for extracts using 100% ethanol. The quantity of TFC tends to increase as the solvent’s polarity decreases, but the highest and the weakest polarity is also not beneficial to increase the TFC. The decline in TFC with higher water content suggests limited flavonoid solubility in aqueous media [28]. It is evident that flavonoids represent the dominant phenolic group within the fruits of *B. nummularia*. This result, supported by current literature, shows that as a key class of dietary phenolics, flavonoids represent about two-thirds of the total phenolic intake from plant foods [32,33].

### 3.4. Antioxidant Activity of B. nummularia Fruit Extracts

#### 3.4.1. Free Radical Scavenging Assay (DPPH)

The DPPH antioxidant activity of *B. nummularia* fruit extracts varied significantly (*p* < 0.05) with the concentration of the ethanol solvent used (Figure 1D), ranging from 13.38 ± 2.65 mg Trolox/100 g DW to 80.99 ± 3.35 mg Trolox/100 g DW. Among the solvents tested, 80% ethanol extracts (80.99 ± 3.35 mg Trolox/100 g DW) demonstrated the highest DPPH value, with those made with 100%, 50%, and 30% solvents following closely behind. This trend can be explained by the solvent polarity-dependent extraction efficiency of major polyphenols in *B. nummularia*, including caffeic acid, rutin, quercetin 3-β-D-glucoside, protocatechualdehyde, and quercetin derivatives.

The major polyphenols in *B. nummularia* have varying polarities, influencing their extraction efficiency. Caffeic acid (moderately polar) more soluble in intermediate ethanol concentrations (40–70%) [34]; rutin and quercetin 3-β-D-glucoside (polar glycosides) prefer hydroalcoholic mixtures (30–80% ethanol) due to their sugar moieties [35]; 3,4-protocatechualdehyde (polar) extracted well in both water and ethanol [36]. Since 80% ethanol extracts exhibited the highest DPPH activity, this suggests that less polar antioxidants such as free quercetin or other aglycones may dominate the antioxidant profile of *B. nummularia*. Moreover, ethanol disrupts plant cell walls more effectively than water, releasing bound phenolics [37].

#### 3.4.2. Ferric Reducing Antioxidant Power

The FRAP value of *B. nummularia* fruit extracts varied significantly (*p* < 0.05) with the concentration of the ethanol solvent used. The FRAP value for the 80% ethanol extracts (594.27 ± 17.23 mg Trolox/100 g DW) for *B. nummularia* was significantly higher than those of other ethanol concentrations (Figure 1E). In contrast, the lowest FRAP values were exhibited at water, with 455.72 ± 37.29 mg Trolox/100 g DW. This result of FRAP test correlated positively with their total phenolic content (CTPC). Notably, extracts with a decreased water content yielded higher FRAP values. Furthermore, the results of DPPH and FRAP indicated a remarkably high antioxidant potential for *B. nummularia* fruit. The minor discrepancies in the values obtained can be attributed to the distinct mechanisms underlying each assay.

However, the purified polyphenols of *B. nummularia* fruits have high phenolic content (PPB), flavonoid content (PFB), and antioxidant capacity; the PPB and PFB reached 3948.25 ± 18.00 mg GA/100 g DW and 4043.66 ± 19.01 mg RE/100 g DW (Figure 1F). This implied that the TCPC has a high content of mishmash and different solvents significantly affected to the extraction values of TCPC and TFC. The DPPH and FRAP values of PPB were 2091.35 ± 23.67 mg Trolox/100 g DW and 5006.71 ± 33.01 mg Trolox/100 g DW); the antioxidant capacity of PPB demonstrated high antioxidant capacity, which was higher than that pulps of orange, strawberry, kiwi, and baobao fruits [10].

### 3.5. Dynamic Adsorption and Desorption

**Figure 2:**
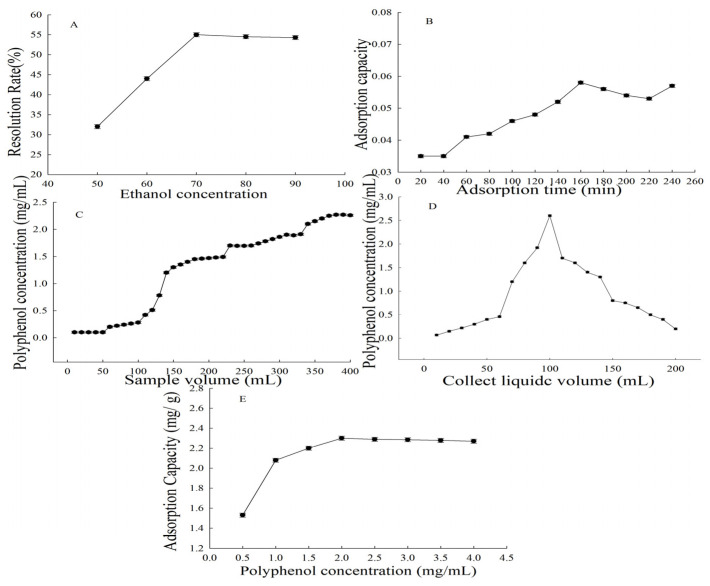
(**A**) Eluent concentration vs. desorption yield; (**B**) adsorption capacity over time; (**C**) dynamic adsorption profile; (**D**) dynamic desorption profile; (**E**) loading concentration vs. adsorption rate.

As shown in Figure 2E, the adsorption capacity of AB-8 macroporous resin increased correspondingly with CPB concentration over the range of 0 to 2.0 mg/m, with the saturation point established as the loading concentration. Kinetic analysis (Figure 2B) indicated that adsorption equilibrium was attained within 150 min. Furthermore, elution with either 70% or 80% ethanol yielded concentrated peaks at 54.91 ± 0.54% and 54.12 ± 0.34%, separately, without trailing and with comparable efficiency (Figure 2A), aligning with the results reported by Wu et al. [38].

As shown in the dynamic adsorption profile (Figure 2C), a CPB extract (2.5 mg/mL) was loaded at 2.0 mL/min and 10 mL fractions were collected for analysis. Polyphenols were first detected in the eluate at a loading volume of 70 mL. The eluate concentration increased thereafter with further loading, attaining 2.45 ± 0.21 mg/mL at 370 mL, which approximated the feed concentration and signified resin saturation.

Following the adsorption saturation of AB-8 macroporous resin using a 2.5 mg/mL CPB solution (Figure 2D), polyphenol desorption was performed with 260 mL of 70% ethanol. The elution flow rate was set at 1.8 mL/min to ensure sufficient contact, thereby achieving an 80% recovery rate when 190 mL of eluent was collected. The target fraction (60–190 mL) was concentrated and freeze-dried, thereby obtaining PPB with a substantially higher purity (71.88 ± 2.74%) than the crude CPB (38.18 ± 2.21%). This resin-based enhancement corroborates Ren et al. [39], where AB-8 resin increased wampee polyphenol purity from 12.51% to 53.8%. The purified percentage of scopolamine reached 84.2% by using D151 macroporous resin [40]. The findings suggest that optimal resin selection is plant-specific, and that AB-8 macroporous resin is appropriate for purifying polyphenols from *B. nummularia* fruits.

### 3.6. Identification of Phytochemicals in B. nummularia Fruits

**Figure 3:**
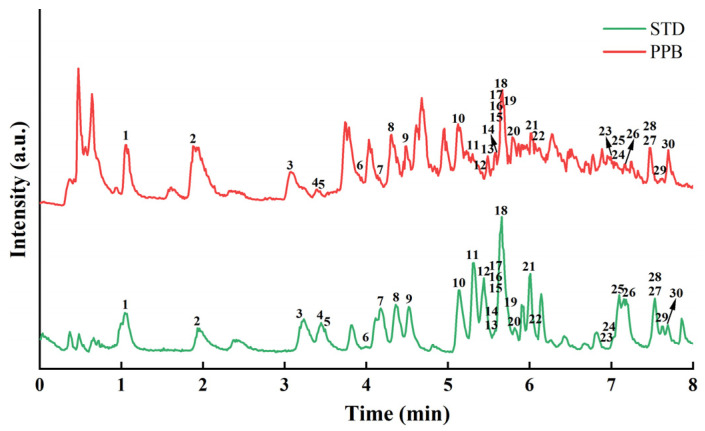
Chromatogram of 30 polyphenol standards and PPB, STD: polyphenol standard; PPB: purified polyphenol of *B. nummularia* fruits.

We employed targeted mass spectrometric quantification, which relied on the accurate m/z and retention time of reference standards for peak assignment. Figure 3 presented the total Chromatogram of purified polyphenol extracts of *B. nummularia* fruits, as analyzed via UPLC-QE. The relative quantification of 30 polyphenol compounds were identified, which comprised 13 phenolic acids (7 benzoic acid and 6 cinnamic acid derivatives), 13 flavonoids, 3 benzaldehydes, and 1 aromatic acid. Among the 13 flavonoids, 2 flavones (23, 27), 3 flavonols (24, 29, 30), 1 flavanone (28), 2 flavan-3-ols (6, 20) and 5 flavonoid glycosides (13, 14, 17, 19, 22) were identified in Table 1.

The predominant polyphenol was caffeic acid at 426.20 ± 0.18 ng/mg, with rutin (223.86 ± 0.21 ng/mg) and quercetin 3-β-D-glucoside (200.31 ± 0.33 ng/mg) being the next most abundant. Phenolic acids such as caffeic acid are key contributors to the overall antioxidant capacity [41]. While caffeic acid, rutin, and quercetin 3-β-D-glucoside exhibit varied chemical architectures, they share common physiological activities in terms of antioxidation, anti-inflammation, anticancer, cardiovascular protection, immunomodulation, and antiviral effects [42,43,44]. These properties make them valuable in the fields of drug development, health supplements, and food additives.

### 3.7. Inhibition Effect of PPB Against α-Amylase

**Figure 4:**
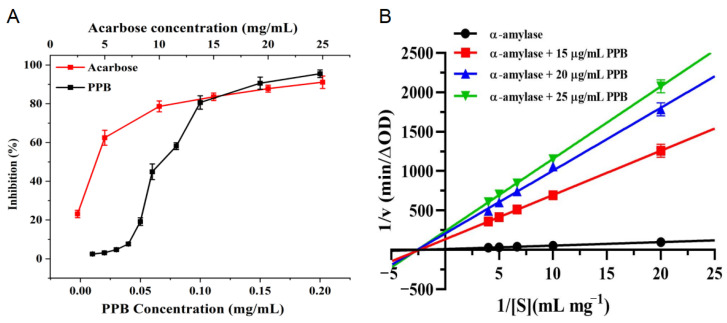
Inhibition against α-amylase activity (**A**) and Lineweaver–Burk plots (**B**).

Both acarbose (positive control) and PPB (phenolic control) displayed dose-dependent α-amylase inhibition (Figure 4A). PPB demonstrated superior activity, achieving a maximum inhibition of 99.40% and an IC_50_ of 69.91 μg/mL, which was considerably lower than that of acarbose (4.42 mg/mL). This aligns with studies reporting caffeic acid’s inhibitory effect (IC_50_ = 3.68–3.69 mg/mL) [45]. However, the findings contradict Shi et al. [46], who observed weak inhibition by caffeic acid using salivary α-amylase. The discrepancy may stem from enzyme source variations (salivary vs. pancreatic) and concentration-dependent effects, as phenolic acids can act as either inhibitors or activators based on concentration [47].

The kinetic pattern demonstrated by the Lineweaver–Burk plot (Figure 4B)—specifically, the lines intersecting in the second quadrant—confirmed a mixed-type inhibition, as evidenced by the increasing V_max_ and decreasing K_m_ [48,49].

### 3.8. Regulation of Postprandial Hyperglycemia by Acute Intake of PPB in Normal ICR Mice

**Figure 5:**
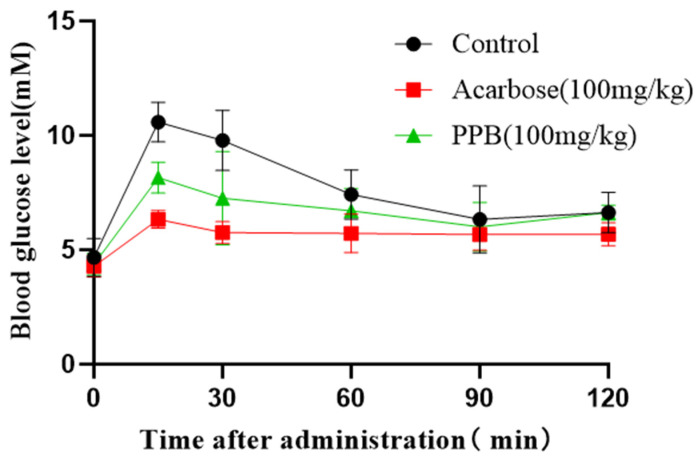
Blood glucose levels after oral administration of acarbose and PPB on postprandial hyperglycemia in normal ICR mice after sucrose challenge.

To evaluate the in vivo α-amylase inhibitory efficacy of PPB, PPB was selected as a candidate inhibitor, owing to its low IC_50_ value—a marker of potent in vitro inhibition that suggests in vivo relevance. An oral sucrose tolerance test in normal ICR mice revealed that the sucrose challenge induced a rapid rise in blood glucose (Figure 5). This postprandial hyperglycemia was markedly suppressed by both acarbose and PPB (100 mg/kg BW), with PPB reducing glucose levels by 13.75 ± 0.87%, demonstrating efficacy comparable to the drug. The acarbose with same dose in regulation of postprandial hyperglycemia was better than that of PPB, but PPB still effectively mitigates postprandial hyperglycemia by inhibiting α-amylase activity. This result is aligned with the effect of the prodelphinidins from Chinese bayberry leaves (BLPs) [50].

This study highlights PPB’s potential as an hyperglycemic agent for its inhibitory effects to α-amylase inhibitors. These findings support further exploration of PPB’s biological applications and market potential. Future research should focus on real-time monitoring of PPB–α-amylase interactions in vivo and optimizing PPB’s binding efficiency to enhance its hypoglycemic effects.

## 4. Conclusions

The highest extraction yield of crude polyphenol of *B. nummularia* (CPB) was shown in 30% ethanol. The highest values of TPC, TFC, DPPH, and FRAP of CPB/PPB were extracted with 80% ethanol solvents. Thus, the study concluded that 80% ethanol is the best solvent for extracting phytochemicals and antioxidants from *B. nummularia* fruits. AB-8 macroporous resin was found to be the optimal material for achieving PPB (71.88 ± 2.74%). Utilizing UPLC-QE, 30 phytochemicals in the *B. nummularia* fruits were identified, and phenolic acids (caffeic acid) and flavonoid glycosides (rutin and quercetin 3-β-D-glucoside) were the main component. PPB suppressed α-amylase (IC_50_ = 69.91 μg/mL), exhibiting non-competitive inhibition, effectively lowered blood glucose levels by 13.75 ± 0.87%, and exerted significant improvement in postprandial hyperglycemia. This study refined the research system for polyphenols from the fruits of *B. nummularia*, clarified the inhibitory activity of PPB against α-amylase, and, for the first time, confirmed that its inhibitory mechanism is non-competitive. From the enzymological perspective, this study elucidated the molecular mechanism by which these polyphenols regulate carbohydrate digestion, thereby enriching research on the anti-diabetic targets of plant polyphenols. The hypoglycemic effect of PPB in vivo was verified via the sucrose loading test, which provides a scientific basis for the high-value utilization of fruits from the genus Berberis and promotes the translation of natural products from basic research to practical applications.

However, ligand–protein docking studies were needed to investigate the interactions between the major bioactive constituents of PPB and α-amylase, with a focus on analyzing key parameters such as binding interfaces and binding affinity. This approach enabled the explication of the molecular mechanism underlying the enzyme inhibitory activity. To corroborate the conformational steadiness of the resulting complexes, molecular dynamics simulations need to be performed. To elucidate PPB’s capacity for reversing diabetic metabolic dysregulation, comprehensive metabolic profiling will analyze serum and hepatic metabolite changes in T2DM animals following treatment. This will facilitate the identification of key pathways corrected by PPB.

## Figures and Tables

**Table 1:** List of the polyphenol compounds of purified extraction of *B. nummularia* fruits (PPB).

Classification		No.	Name	RT (min)	MM	MZ	MS/MS	Contentng/mg
phenolic acids	benzoic acid derivatives	1	Gallic acid	0.98	170.13	169.01	69.03/79.01/107.01/125.02	2.13 ± 0.11
		2	3,4-Dihydroxybenzoic acid	1.86	154.13	153.02	81.03/91.02/108.02/109.03	52.67 ± 0.03
		4	4-Hydroxybenzoic acid	3.41	138.13	137.02	65.04/92.03/93.03	27.93 ± 0.15
		7	Vanillic acid	4.16	168.16	167.03	95.01/108.02/123.01152.01	1.04 ± 0.07
		9	Syringic acid	4.49	198.19	197.05	125.02/138.03/153.02/182.02	2.45 ± 0.16
		18	Salicylic acid	5.69	138.13	137.02	65.04/92.02/93.03	2.38 ± 0.01
		21	Benzoic acid	5.99	122.13	121.03	65.04/77.04/93.03	6.47 ± 0.03
	cinnamic acid derivatives	8	Caffeic acid	4.34	180.17	179.03	93.03/107.01/134.04/135.05	426.20 ± 0.18
		11	Hydroxycinnamic Acid	5.30	164.17	163.04	65.04/93.03/19.05	7.33 ± 0.05
		15	Trans-Ferulic acid	5.63	194.20	193.05	117.03/134.04/149.02/178.03	1.04 ± 0.04
		16	Sinapic Acid	5.65	224.23	223.06	149.02/164.01/179.04/208.04	0.22 ± 0.02
		25	Hydrocinnamic acid	7.10	150.19	149.06	77.04//91.05/105.03	2.65 ± 0.00
		26	Trans-Cinnamic acid	7.19	148.17	147.04	77.04/102.05/103.05	37.30 ± 0.01
flavonoids	flavones	23	Luteolin	7.01	286.25	285.04	151.00/175.04/199.04/217.05	1.76 ± 0.43
		27	Apigenin	7.53	270.25	269.05	117.03/151.00/201.06/225.06	0.27 ± 0.10
	flavonols	24	Quercetin	7.05	302.25	313.03	107.01/151.00/229.05/257.05/273.04	5.40 ± 0.19
		29	Kaempferol	7.63	286.25	285.04	107.01/151.00/227.04/255.03	0.16 ± 0.67
		30	Isorhamnetin	7.68	316.28	315.05	151.00/255.03/271.03/300.03	2.60 ± 0.48
	flavanone	28	Naringenin	7.54	272.27	271.06	107.01/119.05/177.06/151.00	0.83 ± 0.09
	flavan-3-ols	6	Catechin	3.98	290.29	289.07	125.02/179.03/203.07/245.08	0.06 ± 0.15
		20	(+)-Dihydroquercetin	5.89	304.27	303.05	125.02/217.05/257.05/285.04	0.12 ± 0.37
	flavonoid glycosides	13	Rutin	5.55	610.57	609.15	255.03/271.03/300.03/301.04	223.86 ± 0.21
		14	Vitexin	5.61	432.41	431.10	161.02/269.05/283.06/311.06	1.29 ± 0.06
		17	Quercetin 3-β-D-glucoside	5.69	464.41	463.09	255.03/271.04/301.04/300.03	200.31 ± 0.33
		19	Luteoloside	5.72	448.41	447.09	151.00/257.05/284.03/285.04	0.56 ± 0.26
		22	Kaempferol-3-O-glucoside	6.01	448.41	447.09	227.04/255.03/284.03/285.04	0.30 ± 0.27
benzaldehyde derivatives		3	Protocatechualdehyde	3.16	138.13	137.02	64.02/81.03/92.03/108.02	127.75 ± 0.03
		10	Vanillin	5.12	152.16	151.04	81.03/92.03/108.02/136.02	2.88 ± 0.12
		12	Syringaldehyde	5.44	182.19	181.05	108.02/137.02/151.04/166.03	1.06 ± 0.10
aromatic acid		5	Phthalic acid	3.49	166.14	165.02	76.02/93.03/121.03	1.00 ± 0.31

MM: Monoisotopic mass. MZ: Mass-to-charge ratio. MS/MS: Tandem mass spectrometry

## Data Availability

The original contributions presented in this study are included in the article. Further inquiries can be directed to the corresponding authors.
